# Effects of Competitive Exergaming–Based Esports on Older Adults in Hong Kong: Nonrandomized Controlled Pilot Study

**DOI:** 10.2196/77655

**Published:** 2025-12-15

**Authors:** Ka Man Leung, Yuchen Shi

**Affiliations:** 1 Department of Health and Physical Education Education University of Hong Kong Tai Po China (Hong Kong); 2 Department of Business and Strategy Lee Shau Kee School of Business and Administration Hong Kong Metropolitan University Ho Man Tin China (Hong Kong)

**Keywords:** esports participation, exergaming, health promotion, intervention study, older adults

## Abstract

**Background:**

Hong Kong faces a rapidly aging population, with many older adults not meeting recommended physical-activity levels and struggling to maintain long-term exercise adherence. Exergaming offers an accessible, technology-supported way to promote health conditions while providing immediate feedback and task variability among older adults.

**Objective:**

This study aimed to evaluate the effects of an 8-week competitive exergaming intervention on functional fitness, cognition, loneliness, physical activity (PA) enjoyment, and quality of life among community-dwelling older adults in Hong Kong.

**Methods:**

We conducted a pragmatic, nonrandomized, 2-group pilot with community participants allocated to either a competitive exergaming group (EG) or a passive control group (CG). EG attended 16 instructor-led 90-minute sessions playing Fitness Boxing (Nintendo Switch), including head-to-head bouts and a bracketed tournament. Outcomes were assessed at baseline and postintervention. Primary analyses used repeated-measure analysis of covariance (time: pre and post; group: EG and CG) adjusted for age, sex, education, marital status, employment, financial status, and housing. Partial η^2^ was used to quantify effect sizes. Given the pilot nature, *P* values are unadjusted for multiplicity and interpreted cautiously.

**Results:**

A total of 48 older adults completed assessments (EG: n=24; mean age 69.50, SD 4.77 years; CG: n=24; mean age 71.50, SD 6.74 years). Significant group × time interactions favored EG for lower-body strength (30-second chair stand: *F*_1, 48_=12.39; *P*<.001; partial η^2^=0.22), aerobic endurance (2-minute step: *F*_1, 48_=4.89; *P*=.03; η^2^=0.10), and PA enjoyment (Physical Activity Enjoyment Scale: *F*_1, 48_=9.36; *P*<.001; η^2^=0.18). For the Number Comparison Test (processing speed), the group × time interaction was not significant (*P*=.08); however, an exploratory main effect of group indicated higher performance in EG across time (*P*=.04). Executive function (Trail Making Test parts A and B), loneliness, and Short Form-36 Health Survey subscales showed no significant effects.

**Conclusions:**

Competitive exergaming was feasible and produced small to moderate improvements in lower-body strength, aerobic endurance, and enjoyment of PA. Cognitive effects were inconclusive and should not be overinterpreted given the nonrandomized design, passive control, small sample, and multiple outcomes. Future randomized trials with active comparators and longer duration are warranted.

## Introduction

Hong Kong’s aging population poses a considerable public health challenge. The overwhelming majority of older adults in Hong Kong do not meet the minimum standards for physical activity (PA) levels recommended by the World Health Organization (WHO) [1]. According to the Census and Statistics Department of the Hong Kong Special Administrative Region (HKSAR) [2], the number of individuals aged 65 years or older is expected to increase to 2.16 million by 2031 and 2.56 million by 2041. Being physically inactive and having a sedentary lifestyle contribute to physiological, psychological, and cognitive health problems. The financial burden of age-related health issues poses social challenges, including increased pressure on health care and social welfare systems [3]. Due to the effects of net medical inflation, population growth, and aging (assuming service enhancement continues at historical levels), Hong Kong’s recurrent social welfare and health expenditure as a percentage of nominal GDP is projected to increase from US $56.9 billion and US $52.4 billion in 2014 and 2015 to US $523.3 billion in 2041 and US $563.6 billion in 2042. A structural financial deficit may then occur within a decade.

In examining the aging population and its social implications, studies have suggested various types of PA to promote active aging. Theoretically, “exergaming” refers to technology-mediated play that requires bodily movement and yields exercise-like exertion [4]. “Esports” denotes organized competitive digital gaming characterized by structured competition, skill, information technology, and interpersonal interaction [5,6]. We use the term “competitive exergaming” to denote the intersection—exergames delivered with explicit competitive structures (eg, head-to-head play, scoring, and brackets) in supervised, organized sessions. This sits at the amateur and community end of the esports spectrum; esports is commonly defined as organized competitive digital gaming that can range from community tournaments to professional leagues. Our program implemented 2-player bouts throughout training and a double-elimination tournament, thereby satisfying the competitive and organized attributes associated with esports while retaining the physical demands of exergaming. Framing the intervention as competitive exergaming thus clarifies both the active ingredient we are testing and the leisure community context in which it occurred for older adults. In this study, when the competitive mode of play and interactive elements are integrated with physical and cognitive efforts in an exercise gaming environment, we propose competitive exergaming as one form of esports.

The acceptance and recognition of esports are reflected in the International Olympic Committee’s Olympic esports initiatives, which include organizing an esports forum in 2018 and creating the Olympic Esports Series in 2023. Esports is a fast-growing global industry that is particularly prominent in China and Hong Kong [7]. Since 2000, esports has exploded in popularity in China. Chinese esports revenue accounted for more than 18% of global esports revenue (US $13.63 million) in 2018 [8]. Capitalizing on this trend, the HKSAR government proposed esports as a new sector for economic development in 2017 and engaged in several initiatives to promote the development of the esports industry [9]. For example, the HKSAR government provided more than HK$100 (US $12.85) million to Cyberport, a digital technology community in Hong Kong, to promote esports by organizing esports events, empowering the esports industry, developing esports talent, making esports mainstream, and promoting a positive public image [10].

Unlike traditional video gaming, which is often associated with negative consequences, such as excessive screen time, addiction, and sedentary behaviors [11], exergaming offers substantial benefits, including improved physical fitness and range of motion [12], increased stress relief and motivation [13], and enhanced social connectedness and interaction [13]. In this context, competitive exergaming has been studied for its contributions to health and wellness goals among older adults [14]. Monteiro Pereira et al [14] indicated that esports engagement encouraged active lifestyle habits and did not contribute to health problems, contradicting the conclusions of some other studies regarding esports participation, although an association between intense esports practice and mental health problems was observed in certain cases. Moreover, exergames have shown benefits for older adults’ physical function and social outcomes, including improved functional capacity and adherence [15] and reduced loneliness in some contexts [16]. Systematic reviews and meta-analyses indicate exergaming can enhance physical and cognitive outcomes in specific populations and designs [17,18]. Importantly, competitive or gamified features (eg, leaderboards, tournaments) can heighten enjoyment and motivation, which are key determinants of adherence in later life [19,20]. However, few community-based programs have deliberately combined exergaming with formal competition for older adults, leaving a gap in whether “competitive exergaming” offers incremental value for enjoyment and functional gains compared with usual activity. Some esports participants report supplementary off-game physical training, but the on-game activity is typically sedentary; in contrast, exergaming itself is physically active and is the modality evaluated in this study.

To date, the literature examining the effects of esports on health and well-being has focused mostly on young populations, especially young adults (eg, students and collegiate players) [14,21] and competitive esports athletes [22-24]. Moreover, a scoping review investigating the associations between esports participation and health conditions revealed that all participants of the 33 included articles were young adults aged 16-27 years [14]. While the effects of esports on older adults have started to be studied in recent years, participation in competitive exergaming–based esports remains a novel form of PA for older adults and is less investigated. A recent meta-analysis included 47 studies and revealed that exergame training could benefit older adults in multiple aspects of physical functioning, such as balance, upper body strength, lower body strength, aerobic endurance, and gait [25]. However, the competitive and interactive features of esports were absent in these studies. In Hong Kong, Leung and Chu [26] conducted a qualitative study on the perception of esports (eg, e-swimming and e-car racing games) among middle-aged and older adults and demonstrated that the physical benefits (improvements in attentiveness, strategic thinking, and memory function), psychological benefits (cognitive improvements and sense of achievement), and social benefits (social interactions and sense of belonging) of esports participation could be explained using the theory of planned behavior, in which the intentions and attitudes underlying actions influence achievement-driven behavior. Their subsequent qualitative study [27] further examined the esports perceptions and experiences of middle-aged and older adult participants in Hong Kong using a social marketing approach. That study hypothesized that a community-based esports intervention meeting the “five Ps” criteria (product, price, promotion, place, and people) would encourage older adults to participate in esports, increase their PA levels, and improve their health. Although the video games used in those 2 studies were equipped with competitive and interactive elements, the seated, sedentary mode of play meant they could not be considered exergaming-based esports. Based on the aforementioned studies, a clear research gap exists in examining the benefits of competitive exergaming–based esports participation on older adults, including its effects on physical and psychological health. Moreover, previous studies have suggested that older adults could gain a greater sense of enjoyment by engaging in competitive forms of PA [28].

To encourage active aging, this study implemented a competitive exergaming–based esports intervention embedded with competition elements for older adults. This study investigated the effects of competitive exergaming–based esports participation on the physical fitness, cognitive function, and selected psychological attributes (ie, loneliness, PA enjoyment, and quality of life [QoL]) of older adults. We hypothesized that competitive exergaming–based esports participation would significantly improve physical function, cognitive attributes, and psychological health in older adults compared to nonparticipation.

## Methods

### Participants

**Figure 1:**
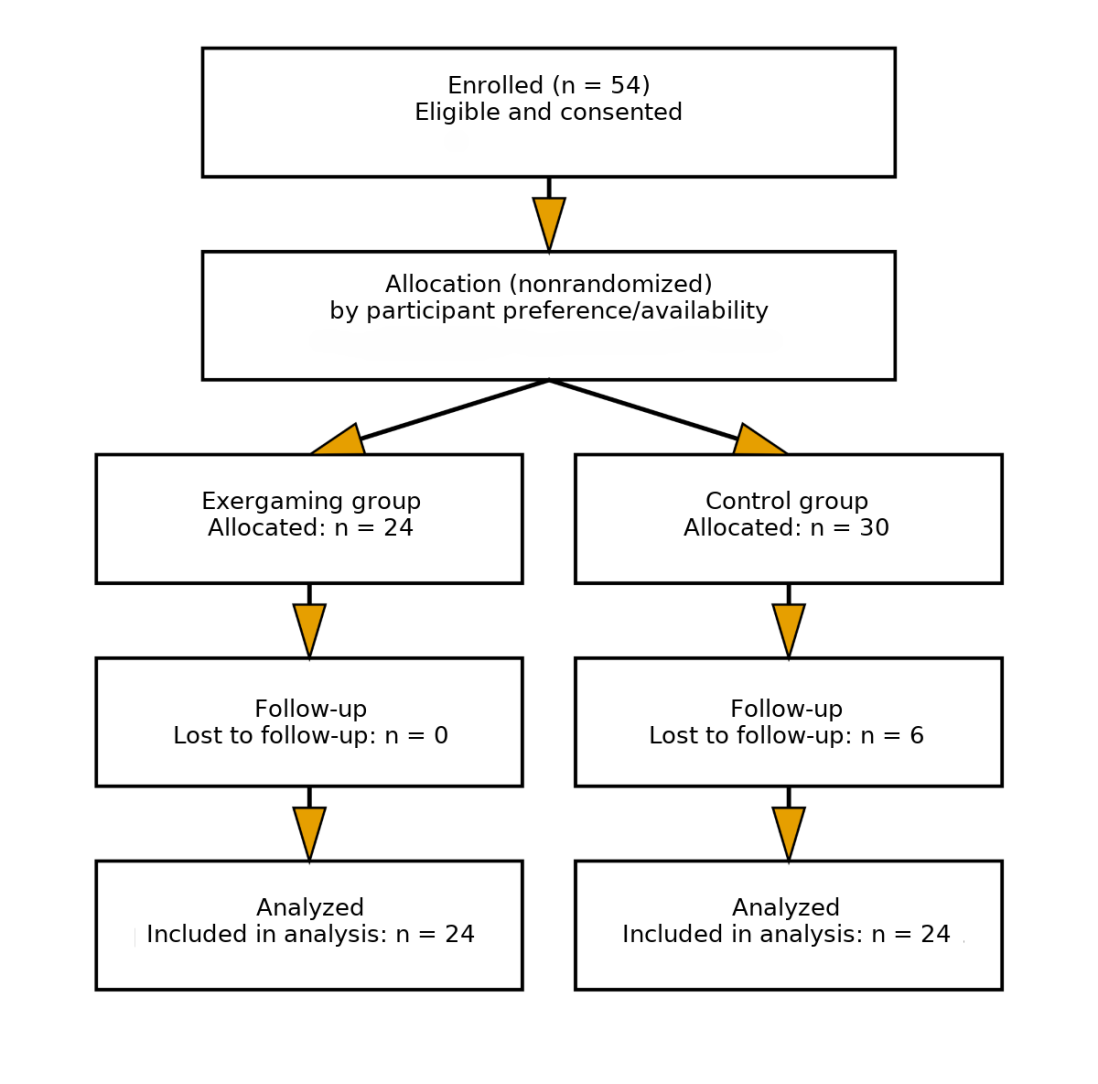
Participant recruitment flowchart.

A convenience sampling strategy was used to recruit participants (Figure 1). Older adults from 2 branches (Kwun Tong and Hing Tin) of a neighborhood older adult care center in Hong Kong were targeted for recruitment between June and August 2021. All recruited participants were assigned to groups stratified by sex and experience with competitive exergaming–based esports activities. The inclusion criteria were (1) being aged 60 years or older, (2) living independently in the community, (3) having no cognitive impairment, (4) not participating in a structured PA program in the 6 months preceding the study (ie, not attending supervised exercise, sport, or fitness classes ≥1 per week for ≥30 minutes), which was confirmed with a brief screening questionnaire at enrollment, and (5) earning passing scores on the Timed Up-and-Go Test [29] and the Abbreviated Mental Test [30]. Participants were required to complete the Timed Up-and-Go Test within 20 seconds. A minimum score of 6 points on the Abbreviated Mental Test was required to qualify for this study. Participant evaluations were carried out by research assistants from The Education University of Hong Kong, who received a 1-day training at the university 1 month prior to recruitment and evaluation. All research assistants were instructed to fully understand the meaning of each question for the measures used in this study. They were responsible for answering any questions raised by participants when completing the questionnaire in both the pretest and posttest.

### Measures

#### Functional Fitness

Functional fitness was evaluated using the Senior Fitness Test developed and validated by Rikli and Jones [31]. The Senior Fitness Test assesses older adults’ ability to perform normal daily activities independently. The test comprises 6 parts. First, the back scratch test measures the distance between the middle fingers of both hands when extended, one over the shoulder and one up the back. Each participant completed 2 trials, and the best distance was recorded. Second, a chair sit-and-reach test requires participants to sit on the edge of a chair with one leg bent and the other leg extended straight in front. The test measures the distance between the extended middle finger and the tip of the toe of the extended leg. Each participant completed 2 trials for each leg, and the best distances were recorded. Third, an 8-foot up-and-go test involved participants standing from a seated position, walking as quickly as possible around a cone placed 8 feet away, and returning to their seats. Each participant completed 2 trials, and the shortest time to finish was recorded. Fourth, a 30-second chair stand test requires participants to repeatedly stand up from a fully seated position as many times as possible within 30 seconds. Each participant completed 2 trials, and the number of stands completed within the time frame was recorded. Fifth, a 30-second arm curl test requires participants to lift a dumbbell with their dominant hand as often as possible within 30 seconds. Each participant completed a practice trial followed by a test trial, and the number of repetitions completed was recorded. Sixth, a 2-minute step test involved participants marching in place, raising each knee to the level of a mark on the wall corresponding to midway between their iliac crest and patella, for 2 minutes. Each participant was allowed a practice trial followed by a test trial, and the number of full steps completed in 2 minutes was recorded. These tests are reliable (intraclass correlation coefficient=0.80-0.98) and valid, as demonstrated in content, construct, and criterion analyses [31].

#### Cognitive Function

To assess cognitive function, 2 tests were administered—the Number Comparison Test (NCT) [32] and the Trail Making Test (TMT) [33]. Both the NCT and TMT have been widely used as cognitive tools for examining older adults’ attention and executive functions [34,35]. The NCT requires participants to identify nonmatching digit pairs by marking an “X” on them within 90 seconds. Scores are calculated by subtracting the number of incorrect markings from the total number of correct markings. The TMT consists of 2 parts. For part A, participants are asked to draw a line connecting the numbers 1 through 25, which are scattered on a page, in ascending order as quickly as possible. Part B requires participants to draw a continuous line alternating between numbers (1-13) and letters (A-L) in ascending and alphabetical order (1-A-2-B, etc). The time taken to complete each part is recorded.

#### Loneliness

Loneliness was measured using a short version of the University of California, Los Angeles (UCLA) Loneliness Scale [36], which has been psychometrically developed and evaluated for older adults in various languages [37]. This scale consists of 8 items evaluating whether participants feel a lack of social contact and assessing their level of loneliness (eg, “I feel isolated from others”). Items are scored on a 4-point scale from 1 (never) to 4 (always), with total scores ranging from 8 to 32. Higher scores indicate a greater level of loneliness. This scale demonstrated satisfactory internal consistency in this study, with a Cronbach α of 0.81.

#### Enjoyment of PA

The level of enjoyment of PA was measured using the Chinese version of the Physical Activity Enjoyment Scale (PACES), which was developed by Chung and Leung [38]. This measure has been validated as a suitable tool to evaluate older adults’ satisfaction with PA [39]. The scale comprises 8 items scored on a 7-point scale from 1 (“I enjoy it”) to 7 (“I dislike it”). A total of 4 of the items (items 1, 4, 5, and 7) are reverse coded. Higher scores indicate greater enjoyment of PA. The scale demonstrated excellent reliability and internal consistency in a previous study (Cronbach α=0.92) [39]. The mean value of the 8 item scores was used as the overall PA enjoyment score.

#### Quality of Life

The Short Form-36 Health Survey (SF-36) is widely used to assess health-related QoL among older adults [40]. In this study, 2 subscales—Social Functioning (2 items) and Emotional Well-being (5 items)—were used. These subscales assess, respectively, motivation to participate in social activities and the frequency of experiencing certain emotional states (feeling nervous, depressed, peaceful, happy, and calm) over the past 4 weeks. Items are rated on a 5-point Likert scale, with higher scores indicating a better state of health. The reliability and validity of the SF-36 have been verified in Chinese participants [41]. In this study, the SF-36 Social Functioning and Emotional Well-being subscales demonstrated acceptable internal consistency, with Cronbach α values of approximately 0.63 and 0.83, respectively [41].

### Intervention

The intervention programs were delivered from July to September 2021 by registered instructors and a research assistant from The Education University of Hong Kong. Sessions were delivered in small groups at 2 neighborhood older adult care centers. Participants trained in pairs using the game’s 2-player mode for head-to-head play. The final 3 sessions implemented a double-elimination bracket. This format operates competitive exergaming in a community and leisure setting for older adults. A registered instructor supervised technique, safety, and progression. All training sessions were carried out at the 2 neighborhood older adult care center branches (Kwun Tong and Hing Tin) in Hong Kong. The program comprised 16 training sessions, with 2 sessions per week, each lasting 90 minutes. Participants trained in pairs using the game’s 2-player mode for head-to-head play. Each 90-minute session comprised a 10-minute warm-up, 70 minutes of game-based practice and competitive bouts (with short rests), and a 5-minute cooldown. The fitness video game Fitness Boxing, developed by Imagineer (Tokyo, Japan) for the Nintendo Switch console, was selected for participants to play. Fitness Boxing was chosen because it requires multiple physical and cognitive skills. This rhythm-based boxing game combines physical exercise with music and requires players to perform movements by following on-screen cues and musical rhythms. Moreover, its unique 2-player mode creates competitiveness between players by comparing the points they score during pairwise competition. Participants used the console’s Joy-Con motion controllers to perform basic boxing moves, including punching, ducking, weaving, and stepping. All exergaming group (EG) participants were asked to take part in the training sessions at the same time. The exercise intensity was designed to range from low to moderate. A Fitness Boxing competition was arranged during the final 3 sessions. After the intervention, participants were expected to have a basic understanding of competitive exergaming–based esports and basic boxing skills. This was a nonrandomized controlled intervention; in line with JMIR Publications’ policy that registration is required for randomized trials and not required for nonrandomized designs, the study was not prospectively registered. Instead, this nonrandomized controlled intervention was reported in accordance with the Transparent Reporting of Evaluations with Nonrandomized Designs (TREND) statement; a completed TREND checklist is provided in Multimedia Appendix 1. The protocol was approved by the Human Research Ethics Committee of The Education University of Hong Kong (reference number 2019-2020-0468) prior to enrollment (the full trial protocol is provided in Multimedia Appendix 2).

### Procedures

We applied a nonrandomized controlled trial design in this study. We selected a nonrandomized design because we anticipated that prospective participants would have strong preferences for participating in the EG over the control group (CG), thus making randomization infeasible. Prior to the intervention, an instructional session was provided to all participants to explain the details of the program. Participants were required to sign consent forms before the commencement of the intervention. After all participants provided written informed consent, they were divided into 2 groups: a competitive exergaming–based esports group (EG) and a control group (CG). A total of 54 older adults participated in our study. All participants measured their height, weight, and body fat percentage using a Tanita body composition analyzer (Model BC-418) and completed the aforementioned fitness tests. EG participants then began the 8-week intervention immediately after the pretest. A qualified boxercise instructor was recruited to teach and train EG participants in the necessary skills (ie, straight punch, hook, uppercut, mixed punches, weaving, and stepping) for playing the game, with assistance from a research assistant. Finally, a Fitness Boxing competition was held in the final 3 sessions using a double-elimination bracket. After the 8-week intervention, all participants completed the outcome measures again following the same procedures as in the pretest. All tests were conducted at the community centers. CG participants were instructed to maintain their normal daily activities during the study period. Group allocation was not randomized. Participants’ preferences and practical considerations were taken into account; those keen to join the exergaming program were allocated to the EG on a first-come, first-serve basis. Once the EG vacancies were fully filled, the remaining participants were served as the CG.

### Statistical Analysis

We conducted repeated measures analysis of covariance (ANCOVA) for each outcome, with time (baseline, post) as the within-participant factor and group (exergaming vs control) as the between-participant factor. We included the following covariates to adjust for baseline imbalances: age, sex, education, marital status, employment status, financial status, and housing. The group × time interaction was the effect of primary interest. With 2 time points, the sphericity assumption is not applicable. We examined model assumptions (normality of residuals, homogeneity of variances, and homogeneity of regression slopes) and observed no material violations. Analyses were run in IBM SPSS Statistics (version 27) using the general linear model (repeated measures) procedure, type III sums of squares, α=.05, and partial η^2^ as the effect-size index.

### Ethical Considerations

Data collection was approved by the Human Research Ethics Committee of The Education University of Hong Kong (reference number 2019-2020-0468). Outcome assessors and data entry staff were blinded to treatment allocation. To maintain blinding, the research assistants who conducted the preintervention and postintervention assessments were not involved in delivering the intervention and were not informed of participants’ group assignments. Participants were instructed not to reveal their group to the assessors. Similarly, data entry was performed using coded identifiers that did not indicate group membership. All participants provided written informed consent and were assured that their personal information would remain confidential. Participants were compensated at the end of the study with a HK $50 (US $6.43) supermarket voucher. This study adheres to TREND guidelines; a completed TREND checklist is provided in Multimedia Appendix 1.

## Results

Participants were asked to complete the questionnaire after completing the intervention program. All sociodemographic questions were self-generated. A total of 54 older adults participated in our study (n=24 in EG; n=30 in CG). However, 6 participants did not complete the posttest; data from the remaining 48 participants (n=24 in EG and n=24 in CG) were included in our analysis. We used complete-case analyses without imputation; intention-to-treat analysis was not performed and is acknowledged as a limitation. A post hoc power analysis showed that this sample size provided 80% power at *P*<.05 to detect the intervention’s average effect size of partial η^2^=0.14. Participant sociodemographic details are presented in Table 1.

**Table 1:** Sociodemographic characteristics of participants.

Characteristic		Esports (n=24)	Control (n=24)
Age in years, mean (SD)		69.50 (4.77)	71.50 (6.74)
Height (cm), mean (SD)		155.63 (8.61)	158.70 (7.13)
Weight (kg), mean (SD)		54.12 (10.90)	57.61 (9.49)
BMI (kg/m^2^), mean (SD)		22.18 (3.21)	22.85 (3.40)
**Sex, n**			
	Female	6	6
	Male	18	18
**Education, n**			
	Primary	3	2
	Secondary	18	12
	Tertiary or above	3	10
**Marriage, n**			
	Married	5	6
	Single	14	16
	Cohabitation	5	2
**Employment, n**			
	Housewife	3	3
	Retired	21	21
**Financial status, n**			
	Low	8	4
	Medium	15	18
	High	1	2
**Housing types, n**			
	Private housing	22	19
	Home ownership scheme housing	2	5

The mean and SD values for physical fitness, cognitive function, and psychological measures at pretest and posttest are presented in Table 2. A total of 48 older adults (n=24 in EG and n=24 in CG) completed the study. There were no significant differences between the EG and CG in any outcome measure at baseline (all *P*>.05). A repeated-measures ANCOVA was conducted to examine group differences from pretest to posttest. In this model, time (pretest vs posttest) was treated as a within-participant factor and group (EG vs CG) as a between-participant factor. In addition, all participant demographic characteristics, including age, sex, education level, marital status, employment status, financial status, and housing type, were included as covariates to control for potential confounding influences. This approach ensured that any group differences at posttest were adjusted for initial differences and background factors. Table 2 is descriptive and shows the unadjusted means (SD) for each outcome at baseline (T1) and postintervention (T2) for both groups (exergaming vs control). An independent samples 2-tailed *t* test was conducted to indicate whether the groups were different before the intervention.

**Table 2:** Summary of measures at baseline (T1) and postintervention (T2).

			T1				Esports (n=24)				Control (n=24)		
			*t* test (*df*)		*P* value		T1 (pre), mean (SD)		T2 (post), mean (SD)		T1 (pre), mean (SD)		T2 (post), mean (SD)
**Functional fitness**													
	Back scratch—left (cm)	0.44 (47)		.66		–1.33 (10.69)		–1.48 (9.42)		–2.69 (10.80)		–2.35 (10.70)	
	Back scratch—right (cm)	0.55 (47)		.58		4.32 (7.64)		3.25 (7.55)		3.06 (8.17)		2.54 (7.22)	
	Chair sit-and-reach—left (cm)	0.73 (47)		.47		4.70 (11.47)		5.40 (12.35)		2.44 (10.05)		6.99 (9.33)	
	Chair sit-and-reach—right (cm)	0.67 (47)		.51		4.89 (10.95)		6.52 (10.91)		2.85 (10.24)		7.23 (9.89)	
	8-foot up-and-go (s)	–0.99 (47)		.33		5.94 (1.12)		4.96 (0.98)		6.31 (1.45)		5.36 (1.36)	
	Chair stand-up (frequency)	–1.11 (47)		.27		14.08 (3.91)		19.79 (5.37)		15.46 (4.69)		16.38 (5.07)	
	Arm curl (frequency)	–1.22 (47)		.23		10.58 (24.01)		20.46 (7.49)		16.71 (5.35)		19.71 (6.24)	
	2-minute step (frequency)	–0.70 (47)		.49		82.38 (22.80)		96.79 (20.69)		87.00 (22.99)		88.71 (17.38)	
**Cognitive function**													
	Number comparison (s)	–0.41 (47)		.68		31.13 (9.44)		33.00 (10.62)		32.25 (9.31)		38.21 (8.09)	
	TMT^a^ part A (s)	–0.14 (47)		.89		74.46 (33.64)		61.18 (15.36)		75.76 (30.03)		71.56 (30.15)	
	TMT part B (s)	–0.80 (47)		.43		44.58 (19.42)		45.24 (18.35)		49.36 (21.85)		45.76 (17.36)	
	ULS-8^b^ (score)	0.77 (47)		.45		1.91 (0.54)		1.89 (0.50)		1.79 (0.54)		1.69 (0.50)	
	PACES^c^ (score)	–1.17 (47)		.25		5.26 (0.85)		5.74 (0.85)		5.59 (1.09)		5.44 (1.10)	
**Quality of life**													
	Social functioning (score)	0.85 (47)		.40		78.56 (10.23)		80.45 (9.95)		80.12 (9.87)		79.67 (10.14)	
	Emotional well-being (score)	–1.10 (47)		.28		82.34 (8.76)		83.12 (8.45)		81.98 (9.12)		81.45 (9.00)	

^a^TMT: Trail Making Test.

^b^ULS-8: University of California, Los Angeles Loneliness Scale.

^c^PACES: Physical Activity Enjoyment Scale.

All inferential tests reported in Table 3 are adjusted for age, sex, education, marital status, employment status, financial status, and housing. In particular, lower-body strength (30-second chair stand test) improved significantly more in the EG than in the CG (group × time: *F*_1, 48_=12.39; *P*<.001; partial η^2^=0.22). Similarly, aerobic endurance (2-minute step test) showed a greater improvement in the EG compared to the CG (*F*_1, 48_=4.89; *P*=.03; η^2^=0.10), again favoring the EG.

**Table 3:** Summary of repeated-measures (group × time) analysis of covariance controlling for confounders.

			Esports, mean (SD)		Control, mean (SD)	Group (*P* value)		T1 (baseline), mean (SD)		T2 (postintervention), mean (SD)		Time (*P* value)		Group × time (*P* value)
**Functional fitness**														
	Back scratch—left (cm)	1.73 (1.99)		1.55 (2.04)		.95	1.66 (1.47)		1.62 (1.49)		<.01		.62	
	Back scratch—right (cm)	3.49 (1.49)		3.34 (1.53)		.95	3.80 (1.15)		3.03 (1.08)		.049		.61	
	Chair sit-and-reach—left (cm)	4.95 (2.09)		4.81 (2.09)		.96	3.57 (1.56)		6.19 (1.56)		.38		.15	
	Chair sit-and-reach—right (cm)	5.62 (2.02)		5.12 (2.02)		.86	3.871 (1.56)		6.88 (1.50)		.93		.29	
	8-foot up-and-go (s)	5.58 (0.21)		5.71 (0.21)		.65	6.12 (0.16)		5.16 (0.15)		.83		.80	
	Chair stand-up (frequency)	16.74 (0.87)		16.11 (0.87)		.62	14.77 (0.62)		18.08 (0.75)		.06		<.01	
	Arm curl (frequency)	18.08 (1.05)		18.29 (1.00)		.89	16.02 (0.79)		20.35 (0.98)		.52		.26	
	2-minute step (frequency)	89.37 (3.89)		88.06 (3.89)		.82	84.69 (3.34)		92.75 (2.81)		.86		.03	
**Cognitive function**														
	Number comparison (s)	31.24 (1.59)		36.05 (1.59)		.04	31.69 (1.26)		35.60 (1.24)		.66		.08	
	TMT^a^ part A (s)	69.92 (4.19)		71.56 (4.19)		.79	75.11 (3.85)		66.37 (2.68)		.77		.13	
	TMT part B (s)	46.83 (2.51)		45.64 (2.51)		.74	46.97 (2.43)		45.5 (2.06)		.32		.68	
	ULS-8^b^ (score)	1.90 (0.10)		1.75 (0.10)		.34	1.85 (0.08)		1.79 (0.07)		.66		.62	
	PACES^c^ (score)	5.47 (0.18)		5.55 (0.18)		.77	5.43 (0.13)		5.59 (0.14)		.15		<.01	
**Quality of life**														
	Social functioning (score)	79.25 (9.80)		80.40 (10.05)		.48	81.10 (9.50)		79.50 (10.20)		.08		.75	
	Emotional well-being (score)	83.50 (8.30)		82.10 (8.95)		.30	84.20 (8.10)		81.55 (9.05)		.16		.70	

^a^TMT: Trail Making Test.

^b^ULS-8: University of California, Los Angeles Loneliness Scale.

^c^PACES: Physical Activity Enjoyment Scale.

The intervention also had a positive effect on participants’ enjoyment of PA. There was a significant group × time interaction for PA enjoyment (PACES score: *F*_1, 48_=9.36; *P*<.001; η^2^=0.18), indicating a higher enjoyment of PA after the program for the EG relative to the CG. Besides, the repeated measures ANCOVA also noted the effect of group was significant of EG on cognitive functioning, measured by the NCT (*F*_1, 48_=4.51; *P*=.04; η^2^=0.09).

In addition to these interaction effects, significant main effects were observed for certain measures. Upper-body flexibility (back scratch test) improved over time in both groups, as evidenced by significant main effects of time for both the left shoulder reach (*F*_1, 48_=10.61; *P*<.001; η^2^=0.20) and right shoulder reach (*F*_1, 48_=4.07; *P*=.049; η^2^=0.09). This indicates that shoulder flexibility increased from pretest to posttest for participants overall. The group × time interaction for the NCT was not statistically significant (*P*=.08); thus, there is no evidence that the intervention improved NCT performance more than the control over time. An exploratory main effect of group (*P*=.04) indicated that, when averaging across time points, EG participants performed better than CG participants; the time main effect was not significant (*P*=.66). Given multiple outcomes and the pilot design, this group difference should be interpreted cautiously and not as proof of a cognitive benefit attributable to the intervention.

No other outcome showed a statistically significant between-group difference or interaction effect. Upper-body strength (30-second arm curl test) did not significantly differ between groups at posttest. The 8-foot up-and-go test (agility and dynamic balance) improved modestly in both groups, but the group × time interaction was not significant, indicating similar gains for EG and CG. Likewise, there were no significant group differences in lower-body flexibility (chair sit-and-reach test) aside from the overall time effect noted in the back scratch, and no between-group improvements were observed in BMI. In terms of cognitive function, executive function as assessed by the TMT (parts A and B) did not differ significantly between the EG and CG (no significant group or interaction effects, *P*>.05 for both parts). For psychological outcomes, loneliness scores (UCLA Loneliness Scale) decreased slightly in both groups but showed no significant group difference or interaction (*P*>.05). Similarly, none of the SF-36 quality-of-life subscales showed a significant improvement or between-group difference after the intervention (all *P*>.05).

## Discussion

### Overview

This study investigated the effects of an 8-week competitive exergaming–based esports intervention on physical fitness, cognitive functions, and psychological attributes among community-dwelling older adults. Our findings revealed that the EG scored higher than the CG on the following four outcome measures: (1) lower-body strength (chair stand test), (2) aerobic endurance (2-minute step test), (3) cognitive functioning (NCT), and (4) PA enjoyment (PACES). However, no differences were observed between groups in flexibility, upper-body strength and endurance, BMI, or loneliness. These results partially support the hypothesis that greater lower-body strength, agility and dynamic balance, aerobic endurance, cognitive functioning, and PA enjoyment would be observed in competitive exergaming–based esports participants compared to their peers who did not engage in competitive exergaming–based esports.

Despite the statistically significant improvements observed in the EG, the magnitude of changes was modest in absolute terms. The effect sizes for the significant group differences were in the small to moderate range (partial η^2^≈0.10-0.22). For example, on the chair stand-up test, the EG performed only a few more repetitions on average than the CG at posttest. Similarly, the gain of about 14 steps in the 2-minute step test for the EG, while significantly higher than the CG’s change, represents a moderate improvement. These modest effect sizes suggest that, from a clinical perspective, the benefits, although measurable, were not very large over the 8-week period. Caution is therefore warranted in interpreting the clinical significance of the findings. It is possible that longer or more intensive training would be required to translate these statistical improvements into more pronounced functional gains. Nonetheless, even small improvements in strength or endurance can be meaningful in an older population, as they may contribute to better functional capacity and reduce the risk of disability over time.

### Improvements in Physical Health

Significant improvements in lower-body strength and aerobic endurance were observed in the EG. These improvements were attributed to the physical intensity required to play Fitness Boxing during the 8-week intervention. Instructors taught various skills, including different punches (hooks, uppercuts, and mixed combinations), as well as ducking, weaving, and stepping, and participants practiced these skills throughout the intervention. Specific training sessions focusing on advanced skill combinations were organized to gradually increase the intensity of practice. The total activity time in this competitive exergaming–based esports program exceeded the WHO’s PA guideline of a minimum of 150 minutes of moderate-intensity PA per week [1]. This level of activity likely accounts for the observed improvements in physical attributes among older adults in the EG. Moreover, our results align with those of a systematic review indicating that older adults who played exercise-based or motion-sensing games exhibited improvements in physical functions [42]. Another study on the effects of exergames and video game training on older adults’ physical functioning also demonstrated significant enhancements in lower-limb strength and balance among participants in an exergame training group [42]. Our findings are consistent with those of research using exergames involving motor function and balance in rehabilitation, suggesting the positive effects of competitive exergaming–based esports participation on promoting older adults’ physical health [43]. In this study, **agility** (8-foot up-and-go) improved to a similar extent in both groups, suggesting a general practice or learning effect on this quickness or balance task rather than a unique benefit of the intervention. Notably, our intervention did **not** significantly improve upper-body strength or endurance as measured by the arm curl test. This outcome is understandable because the Fitness Boxing game, while it involves arm movements, does not provide progressive resistance to build muscle strength. Participants were punching the air rather than lifting weights or hitting a heavy bag, so the stimulus for upper-body strength gains was minimal. Without targeted resistance training for the arms, even an active game is unlikely to produce measurable improvements in arm strength. Likewise, measures of flexibility (eg, chair sit-and-reach) did not differ between groups; any small gains in flexibility (such as the improved back scratch test scores over time) occurred in both groups, likely due to general daily activities or repeated testing. In summary, the exergame program proved effective for improving lower-body physical fitness components that are linked to the game’s activity profile, whereas fitness components not strongly engaged by the game (upper-body strength and flexibility) showed no differential change.

### Unchanged in Cognitive Functions

Our null findings on the Trail Making Test performance are consistent with the task-specificity of executive training. Fitness Boxing heavily loads visuomotor tracking and reaction speed but offers little explicit set-shifting or working-memory updating—the core demands of TMT part B. Executive improvements from exergames are most evident when games embed dual-tasking, inhibition, and switching mechanics (eg, cybercycling with virtual competitors or exergames designed with executive challenges), whereas physically engaging but cognitively simple titles seldom transfer to TMT-like outcomes [44-46]. This pattern is also reflected in network meta-analytic evidence showing that interactive, cognitively stimulating video game formats outperform low–cognitive-load comparators on processing speed and global cognition in older adults [47].

Dose and population also matter. Meta-analyses in clinical cohorts (mild cognitive impairment or dementia) report larger cognitive effects, particularly when interventions extend beyond ~12 weeks and incorporate targeted cognitive components, whereas short, low–cognitive-load programs in cognitively healthy samples often yield small or null changes [48,49]. Our 8-week esports program, centered on a reflex-oriented title, therefore may have been insufficiently long or specific to move executive outcomes. A recent randomized controlled trial in dementia further underscores this point—exergaming with explicit cognitive engagement produced superior executive gains versus aerobic activity alone [50].

Finally, measurement issues can mask subtle treatment effects. Short-interval retesting on neuropsychological batteries, including TMT variants, produces robust practice (retest) effects that complicate detection of small true changes without large samples or reliable-change methods [51,52]. Together, these literatures align with our finding of no cognitive benefit: without explicit executive-load mechanics, adequate duration, and sensitive analytic approaches, exergaming may leave TMT-like measures unchanged in healthy older adults. These findings are consistent with those of other studies showing that exergame training positively affects older adults’ cognitive functioning [42,53]. For instance, older adults trained using Nintendo Wii exergames demonstrated substantial improvements on the NCT and TMT parts A and B [53]. Moreover, Hou and Li [42] found that an exergaming training intervention enhanced cognitive functioning—specifically verbal memory, top-down attention, and learning efficiency—in older adults. One plausible explanation for the significant improvements in cognitive function observed in our study is the complexity of the exergaming. By providing dynamic game content that combines both cognitive and physical exercises during play [53], the Nintendo Switch Fitness Boxing game exemplified a cognitively demanding exergame, as it required players to continually monitor the screen and adjust their movements to respond appropriately. This complicated play style requires significant cognitive effort and is likely responsible for the cognitive benefits observed in older adults. Neurological mechanisms may also underlie these cognitive improvements. In our study, the exergame’s combination of physical and mental demands (often referred to as “dual-task” training) could have strengthened neural processes related to quick decision-making and visuomotor coordination, leading to the observed improvement in the number comparison task. Engaging in dual-task activities that simultaneously challenge the mind and body can activate prefrontal executive function networks and promote visual-motor integration, leading to improved cognitive processing. Over time, such stimulation may induce neuroplastic changes that enhance information processing speed and attention in older adults [54,55]. In other words, the combined mental and physical challenge of exergaming likely strengthened neural pathways associated with executive function in our participants, contributing to their gains in cognitive performance.

### Improvements in PA Enjoyment

One of the noteworthy outcomes of this study is the significant increase in enjoyment of PA reported by the EG. Participants in the EG had a higher postintervention PACES score, reflecting that they found the PA intrinsically more enjoyable after engaging in the Fitness Boxing program. These results are consistent with studies using other exercise-based video games on different exergaming technology platforms, such as Nintendo Wii [56] and Microsoft Kinect [42], as well as a study using interactive video dance games [56]. Lee et al [19] reported that enjoyment is the primary reason individuals play exergames. Unlike traditional video games, where participants sit and merely manipulate a controller, Fitness Boxing is an exercise-based game that requires players to be active by engaging in a boxing match against human or computer opponents—an enjoyable form of PA. Additionally, the Fitness Boxing competition for EG participants at the end of the program may explain the increase in enjoyment. This suggestion is consistent with the findings of Vorderer et al [57] regarding factors affecting video game enjoyment, which indicated that competition is a key element driving players’ enjoyment. Therefore, the Fitness Boxing competition in the final 3 sessions likely enhanced the overall experience and contributed to the players’ enjoyment of PA. On the other hand, the intervention’s impact on other psychological measures was limited. Feelings of loneliness did not significantly change in the EG compared to the CG. Initially, we hypothesized that participating in a group-based esports activity might enhance social connectedness and reduce loneliness. While participants did interact with each other and the instructors during the program, the duration (8 weeks) and frequency of meetings (twice weekly) may not have been sufficient to produce a measurable reduction in loneliness, especially if participants already had moderate or low loneliness at baseline. It is also possible that more explicit social components (eg, team-based play or off-session social gatherings) are needed to influence loneliness through an exercise program.

Similarly, there were no observable improvements in health-related QoL as measured by the SF-36. QoL is a broad outcome that encompasses physical, emotional, and social well-being. Given the relatively short intervention and the fact that many QoL domains (eg, general health perceptions, social functioning) were not directly targeted by our program, it is not surprising that SF-36 scores remained stable. Often, significant changes in QoL in older adults are reported in longer-term interventions or those that produce large changes in functional ability or mental health. In our study, the improvements in physical function, while meaningful, may not have been large enough over 8 weeks to translate into perceptible changes in participants’ day-to-day QoL.

### Limitations and Future Research

This study is the first to implement a competitive exergaming–based esports intervention program to assess physical fitness, cognitive functions, and psychological attributes among community-dwelling older adults. Nevertheless, the study is not without limitations. First, participants were recruited through convenience sampling from only 2 community centers for older adults, and most participants lived near these centers and preferred to participate with friends from the same center. This situation may have introduced bias in participant recruitment. Unmeasured factors (such as motivation or social engagement levels) could have influenced who chose to join the esports program. This nonrandom sampling and group allocation could have led to selection bias and potentially inflated the intervention effects. Because group allocation followed participant preference on a first-come, first-served basis, the EG may have comprised more motivated or socially engaged individuals at baseline. This nonrandom allocation increases the risk that unmeasured factors, rather than the intervention, contributed to observed differences. The findings are framed as preliminary signals requiring confirmation in randomized trials. Second, the predominance of female participants (36/48, 75%) limits the generalizability of the results. Notably, enjoyment of PA may differ by sex; with so few male participants, we could not assess whether older male participants derive similar enjoyment and benefits from exergaming as older female participants. Future research should use random sampling from diverse locations to enhance generalizability. Expanding the sample size and including multiple centers would also improve generalizability. Third, no follow-up assessments were conducted to monitor older adults’ continued competitive exergaming–based esports participation and any changes in outcomes over time. Because competitive exergaming–based esports is still a novel form of PA for older adults in Hong Kong, researchers should conduct longitudinal studies with follow-up assessments to examine retention in competitive exergaming–based esports participation and the sustainability of its health effects. Fourth, this study only examined the influence of competitive exergaming–based esports on older adults’ health, and the mechanisms behind these benefits (eg, the role of competition) remain unexamined; future studies should investigate how the competitive nature of esports contributes to health outcomes. The fifth limitation is that the control group did not engage in any alternative activity; they were instructed to continue their usual routines, which led to a Hawthorne effect. While this allows us to attribute changes to the intervention, it also means the social interaction and novelty were only experienced by the EG. These nonspecific factors can elevate EG members’ enjoyment (and sometimes performance) independent of the specific active ingredients of exergaming. Besides, participants may behave differently if they understand that they are being observed. In this study, both groups were stationed in different centers where they were members. Participants therefore felt more comfortable interacting in the familiar environment and timing of their own older adult care center, which may reduce their awareness of being observed and hence minimize the Hawthorne effect. Additionally, outcome assessors were blinded so they were less aware of the research aims and further reduced bias. A more rigorous design in the future might use an active control group (eg, a traditional exercise program or a social activity group) to account for placebo or expectancy effects and to isolate the specific contribution of the exergaming format. Another limitation could be the relatively small sample size (n=48), limiting the statistical power to detect smaller effects and preventing any detailed subgroup analyses (eg, comparing outcomes for male vs female participants). A larger sample would provide more confidence in the stability of the results and allow subgroup analyses (eg, by age range or fitness level). Moreover, we did not implement intention-to-treat procedures, which is a limitation; estimates could be affected by differential loss to follow-up (6/30 in CG and 0/24 in EG). Future randomized controlled trials should prespecify and apply intention-to-treat analyses based on the intervention design in this study. Finally, with many end points, the risk of type I error is nontrivial. We report unadjusted *P* values and treat findings as hypothesis-generating. Future confirmatory trials should prespecify a primary end point and control multiplicity.

### Conclusion

In this nonrandomized pilot, competitive exergaming produced small to moderate gains in lower-body strength, aerobic endurance, and PA enjoyment, while cognitive effects were inconclusive. This examination of the benefits of competitive exergaming–based esports participation on older adults’ physical and psychological health outcomes has several practical implications. The improvement in lower-body strength can contribute to better functional mobility. Even a small increase in the ability to rise from a chair or perform repeated standing movements can enhance an older adult’s independence in daily activities (eg, making it easier to climb stairs or get up from a low seat without assistance). Improved aerobic endurance, as reflected in the step test, suggests that participants in the exergaming program built greater stamina. This could translate into an increased capacity for walking longer distances, participating in community events, or simply maintaining energy levels throughout the day. Importantly, these physical gains were achieved through a mode of exercise that participants found enjoyable, which addresses one of the common barriers to exercise in older people—lack of interest or motivation. The enjoyment aspect implies that competitive exergaming–based exercise could lead to higher adherence rates if implemented in community centers or senior exercise programs, thereby potentially yielding long-term health benefits beyond the duration of the study. The fact that this was achieved through a fun PA is noteworthy—it suggests a dual benefit of exergaming for both body and mind. Geriatric health practitioners and recreation program planners might consider incorporating exergaming, or “active video gaming,” sessions into their regular offerings for older adults. Such programs can be a novel way to engage older people who might be reluctant to join traditional exercise classes, thereby broadening participation in PA among this population. However, given selection bias, including a passive control and multiple outcomes, these results should be viewed as preliminary. Randomized, adequately powered trials with active comparators and game mechanics that directly load executive processes are warranted.

## Appendix

Multimedia Appendix 1 TREND checklist.

Multimedia Appendix 2 The trial protocol.

## Abbreviations

ANCOVA: analysis of covariance
CG: control group
EG: exergaming group
HKSAR: Hong Kong Special Administrative Region
NCT: Number Comparison Test
PA: physical activity
PACES: Physical Activity Enjoyment Scale
QoL: quality of life
SF-36: Short Form-36 Health Survey
TMT: Trail Making Test
TREND: Transparent Reporting of Evaluations with Nonrandomized Designs
UCLA: University of California, Los Angeles
WHO: World Health Organization
