# Supplementary material for: Effects of Competitive Exergaming–Based Esports on Older Adults in Hong Kong: Nonrandomized Controlled Pilot Study
Source: JMIR Serious Games. 2025 Dec 15;13:e77655. doi: 10.2196/77655 (PMC12750075; doi:10.2196/77655)
Supplement: Multimedia Appendix 2 [file games_v13i1e77655_app2.pdf]

**Reference Number**	2019-2020-0468
**State**	Reply To PI
**User ID**	leungkaman
**Name of Investigator**	LEUNG Ka Man
**Post**	HPE - Assistant Professor
**Telephone Number**	29488470
**Telephone Number**	29488470
**Type of Funding**	Internal Funding
**Level/Category**	University-level
**Title of the Funding Source**	Funding Support to GRF Proposal Rated 3.5
**Project Title**	Esports intervention in healthy older adults in Hong Kong: a pilot study
**Project From**	1/1/2021
**Project To**	12/31/2021
**Area/Field of Research**	health and older adults

Purpose of the Research
2-3 sentences explaining the main goal of the research

- To examine effectiveness of esports intervention program to improve the physical health outcomes of healthy older adults in Hong Kong.

- To examine effectiveness of esports intervention program to improve the psychosocial health outcomes of healthy older adults in Hong Kong.

Target population: 54 older adults (27 in each group)

Participants Criteria:(a) aged 60 years and above; (b) living in the community independently; (c) absence of diagnosed cognitive impairment; (d) no participation in a structured physical activity program preceding the study; and (e) passing score on the Timed-up-and-go test (TUG, Podsiadlo & Richardson, 1991) (less than 20seocnds) and Abbreviated Mental Test (AMT; Chu, Pei, Ho, & Chan, 1995) (score higher than 6).

Venue of data collection: partnered elderly center

Measurements:

Physical health:

Function fitness: Senior fitness test (Rikli & Jones, 2013). The tests will include 30s Chair stand, 30s arm curl, chair sit and reach, back scratch, 8-feet up and go, and hand grip strength.

Psychosocial and cognitive health:

Loneliness: short form UCLA loneliness scale (Xu, Qiu, Hahne, Zhao, & Hu, 2018)

Physical enjoyment: Physical Activity Enjoyment scale – Chinese (Chung & Leung, 2019)

Cognitive function: Trail Making Test (Wang, Zhou, Huang, & Yang, 2018) and Number Comparison Test (Ackerman & Cianciolo, 2000).

Quality of Life: subscale “social function” and “mental health” of 36-item Short-Form Health Survey (SF-36; Hobart, Williams, Moran, & Thompson, 2002)

Procedure:

Participants will be divided to two groups: Esports group (EG) and control group (CG). For those in Esports group, they will participate in an 8-week intervention, with two sessions per week and 90 minutes per session. They will be instructed to play a game using Nintendo switch. During the intervention, the participants in the control group will be instructed to maintain their normal daily activity. All participants in EG and CG will complete questionnaire about demographic information, physical and psychological attributes, and fitness tests before and after 8-week intervention. Estimated time completing the questionnaires and fitness test is about 1 hour.

Does your research involve human participants directly?

Yes

Does your research involve other human data, e.g. secondary data, archival data, etc.?

No

Please fill in the below information about the participants (in groups) involved in your research project including number of participants, backgrounds of the groups and age range, etc.

A. Participants

- No. of participant: 54
- Requirement of participants: :(a) aged 60 years and above; (b) living in the community independently; (c) absence of diagnosed cognitive impairment; (d) no participation in a structured physical activity program preceding the study; and (e) passing score on the Timedup-and-go test (less than 20seocnds) and Abbreviated Mental Test (score higher than 6).

Are there any reimbursements or other incentives to participants?

Yes

Please mention the cost and the form of reimbursements or incentives offered and clarify why they are reasonable)

A HKD$150 supermarket voucher will be given to the participants after intervention in person to acknowledge their contribution to the study

Participants will be recruited through a recruitment talk offered by the research team and by advertisements in the local

**Please explain your way(s) of recruiting your participants and inviting them to join in step-by-step detail.**	neighborhood elderly centers. An information session (e.g., aims and procedures of the intervention) will be delivered to groups of potential participants. Participants will be advised of the confidentiality of personal data and informed that they can voluntarily withdraw from the project at any time without prejudice. Upon agreement from participants, data collection will be conducted in person by a research assistant and trained student helpers after obtaining informed consent.
**If applicable, explain how you will obtain the participants' contact information in detail.**	No. Participants will be contacted by the person-in-charge of the elderly center.
**Does your research project involve organizations other than EDUHK?**	Yes
**Please identify the data collection site(s), and describe how you will obtain consent/ permission from the data collection site(s). If no written consent will be obtained, please state the reasons**	Data will be collected at the centers. A written consent form with the purposes of the study, their involvement and the risk of participant will be given to the participant upon their agreement of participation.
**Are there any relationships between people involved in the recruitment and the participants (such as teacher and students, principal and teachers, nurse and patients)?**	Yes
**State the nature of the relationship, and mention the special precautions which will preserve their rights to decline to join or withdraw from participation once the research has started**	service provider and service recipents. There will be special precautions which will preserve the participants' rights to decline to join or withdraw from participation once the research has started.
**NotAbleToGiveConsent**	No
**b. Will there be any coercion on the part of the investigator?**	No
**c. Will the data collected have any personally identifiable information of living people, such as name, address, ID numbers, etc? (If so, see HREC Operational Guidelines - Part V regarding Confidentiality and Storage of Data)**	No
	No
**e. Will the study collect information regarding sensitive aspects of the research participants' behavior such as drug and alcohol use, illegal conduct, or sexual behavior?**	No
**f. In case the information on the research participants is disclosed, will it reasonably place the research participants at risk of civil or criminal liability or damage the research participants' financial standing, employability or reputation?**	No
**g. Will financial or other inducements (other than reasonable expenses and compensation for time) be offered to research participants?**	Yes
**h. Will deception of research participants be necessary during the study? (If so, explain why deception is necessary. Also, please include information on debriefing procedures)**	No
**i. Will the study involve prolonged and repetitive testing?**	No
**j. Will the study cause psychological stress or anxiety?**	No
**k. Will pain or more than mild discomfort is likely to result from the study?**	No
**l. Are drugs or placebo to be administered to the research participants?**	No
**m. Will the study involve any intervention?**	Yes
**n. Will blood or tissue samples be obtained from research participants?**	No
**o. Will the research involve any DNA work or human embryo or stem cell research?**	No
**p. Will the research participant's identity be disclosed if archived tissue samples or personal / medical / social records are used? If your study is not a medical/clinical research, please choose NA.**	NA
**q. Will you use irradiation or hazardous substances on research participants?**	No
**r. Will the study impinge on the research participants' right to privacy or their personal life?**	No
**s. If you have checked "Yes" to any of the above questions, please provide elaboration below:**	**The target participants is older adult. HKD$50 supermarket voucher would be provide for compensation for their time contributed in the study.**<br><br>**m: participants will take part in a Esport intervention.**<br>No<br>In the Eports group, you will be provided information on how to play the Nintendo Switch game properly. You may experience fatigue or muscle sore. However, these experiences will be short lived and you should have fully recovered in

days. Also, before and after each intervention session, warm up and cool down activities will be done to lower the chance having injuries.
The answers you provide in the study are confidential, which means that only the study investigators will have access to any information you share. Your identity as a participant in this research study will also be kept confidential in any publication of the results of this study. The data will be reported/written in aggregate terms. *Entered data will be stored on a password-protected file and a password-protected computer, while original, anonymized hard copies of the questionnaires will be stored in a locked office until 5 years past publication. Only PI, and his/her research assistants can access the data.*

Attachment

**File**	**Description**
Combined questionnarie (esport intervention) Chi.docx@!@application_vnd.openxmlformats-officedocument.wordprocessingml.document	Data collection form, including questionnaire (if applicable)
hrec_consent_participants_esport pilot test_EngChi_03.08.2020_finalised.doc@!@application_msword	Consent form and Information Sheet for Participants
hrec_consent_organization__esports pilot test_EngChi_03.08.2020_finalised.doc@!@application_msword	Consent form and Information Sheet for Participants
GRF support fund - Esport interventino in older adults_30JUL2020_Carman.docx@!@application_vnd.openxmlformats-officedocument.wordprocessingml.document	Research Proposal
